# High‐Performance Heterostructured Cathodes for Lithium‐Ion Batteries with a Ni‐Rich Layered Oxide Core and a Li‐Rich Layered Oxide Shell

**DOI:** 10.1002/advs.201600184

**Published:** 2016-05-30

**Authors:** Pilgun Oh, Seung‐Min Oh, Wangda Li, Seunjun Myeong, Jaephil Cho, Arumugam Manthiram

**Affiliations:** ^1^ Materials Science and Engineering Program & Texas Materials InstituteThe University of Texas at AustinAustinTX78712USA; ^2^ Department of Energy EngineeringSchool of Energy and Chemical EngineeringUlsan National Institute of Science and Technology (UNIST)689‐798UlsanSouth Korea

**Keywords:** chemical activation, heterostructure, lithium‐rich layered oxide, nickel‐rich layered oxide, surface stabilization

## Abstract

The Ni‐rich layered oxides with a Ni content of >0.5 are drawing much attention recently to increase the energy density of lithium‐ion batteries. However, the Ni‐rich layered oxides suffer from aggressive reaction of the cathode surface with the organic electrolyte at the higher operating voltages, resulting in consequent impedance rise and capacity fade. To overcome this difficulty, we present here a heterostructure composed of a Ni‐rich LiNi_0.7_Co_0.15_Mn_0.15_O_2_ core and a Li‐rich Li_1.2−_ *_x_*Ni_0.2_Mn_0.6_O_2_ shell, incorporating the advantageous features of the structural stability of the core and chemical stability of the shell. With a unique chemical treatment for the activation of the Li_2_MnO_3_ phase of the shell, a high capacity is realized with the Li‐rich shell material. Aberration‐corrected scanning transmission electron microscopy (STEM) provides direct evidence for the formation of surface Li‐rich shell layer. As a result, the heterostructure exhibits a high capacity retention of 98% and a discharge‐voltage retention of 97% during 100 cycles with a discharge capacity of 190 mA h g^−1^ (at 2.0–4.5 V under C/3 rate, 1C = 200 mA g^−1^).

## 1 Introduction

This is an open access article under the terms of the Creative Commons Attribution License, which permits use, distribution and reproduction in any medium, provided the original work is properly cited.

Mn‐based Li‐rich layered oxides Li_1+_ *_x_*Mn_1−_ *_x_*_−_ *_y_*M*_y_*O_2_ (*x* > 0 and M = transition metal) offer high capacities of >250 mA h g^−1^, but are faced with a fundamental challenge of working voltage decline during electrochemical cycling due to a layered to spinel‐like phase transformation, which leads to severe decrease in energy density during operation.^1^, ^2^, ^3^, ^4^ To overcome this problem, many studies have focused on stabilizing the structure with various efforts, such as surface modifications and transition‐metal‐ion substitutions,^2^, ^5^, ^6^, ^7^, ^8^ but none of the efforts could completely eliminate the phase transition. In this regard, new approaches are needed to develop high‐capacity cathodes.

**Figure 1:**
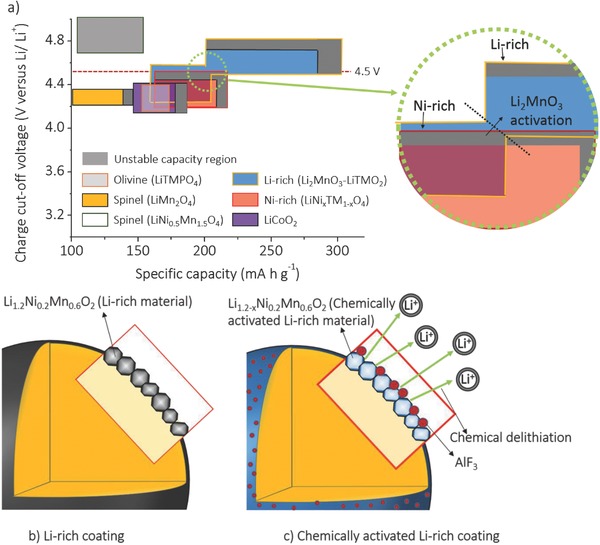
a) Illustration of the specific capacity along with the charge cut‐off voltages of the cathode materials for lithium‐ion batteries. Schematic views of b) the single coating of Li_1.2_Ni_0.2_Mn_0.6_O_2_ and c) the double coating with Li_1.2_Ni_0.2_Mn_0.6_O_2_ and AlF_3_.

**Figure** **1**a shows the practical capacities of various cathode materials for lithium‐ion batteries along with the charge cutoff voltages.^3^, ^9^ Each cathode material has a trade‐off relationship between its capacity and cyclability.^10^ For example, the cyclability decreases as the charge cut‐off voltage and the capacity are increased. The instability of the active materials is caused by two deterioration mechanisms: (i) side reactions of the cathode surface with the electrolyte and (ii) bulk phase transitions.^11^, ^12^ Many studies have recently pointed out that these deteriorations start at the active material surface, and then they extend to the bulk.^6^, ^11^, ^13^, ^14^ Therefore, stabilizing the active material surface is critical to realize good cycle life.

With an aim to increase the capacity, Ni‐rich layered oxides LiNi_1−_ *_x_*M*_x_*O_2_ (*x* < 0.5 and M = transition metal) are drawing much attention recently, but they have to be charged to a higher cut‐off voltages of ≈4.5 V versus Li/Li^+^.^15^, ^16^ Unfortunately, at the high charge voltages, the nickel ions undergo severe side reaction with the electrolyte,^13^, ^17^ resulting in the formation of a thick solid‐electrolyte interphase (SEI) layer, increase in impedance, and decrease in energy density.^10^, ^17^, ^18^ In contrast, the Mn‐based Li‐rich layered oxides show better cyclability than Ni‐rich layered oxides while cycling with a higher cut‐off voltage of ≈4.5 V (Figure 1a),^6^, ^19^, ^20^ implying that the Li‐rich oxides display better surface chemical stability although they show poor structural stability due to the layered to spinel‐like transition. In this regard, a heterostructure composed of Ni‐rich core and Li‐rich shell could offer superior performance by minimizing the surface chemical instability of the Ni‐rich core and the voltage decline problem of the Li‐rich shell.

Accordingly, we present here a novel heterostructure composed of a Ni‐rich LiNi_0.7_Co_0.15_Mn_0.15_O_2_ core and a Li‐rich Li_1.2−_ *_x_*Ni_0.2_Mn_0.6_O_2_ shell, incorporating the strong points of the structural stability of the core and chemical stability of the shell. Furthermore, in order to realize the high capacity as well as the high stability of the shell, a unique chemical activation method is used (Figure 1b,c). The bare Ni‐rich LiNi_0.7_Co_0.15_Mn_0.15_O_2_ is first coated with a Li‐rich Li_1.2_Ni_0.2_Mn_0.6_O_2_ shell layer, and then the Li‐rich shell layer is chemically activated with an AlF_3_ treatment to yield high capacity even with a low charge voltage of 4.5 V.^6^, ^21^, ^22^

## 2 Results and Discussion

**Figure 2:**
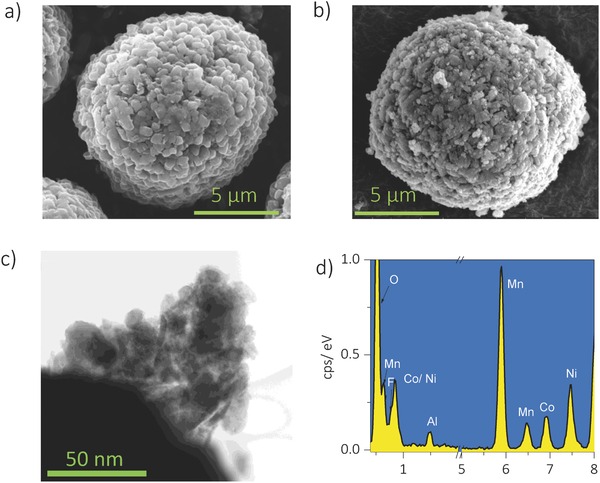
Scanning electron microscopy (SEM) images of a) the bare LNCM and b) 20LNM‐ALF_3_‐coated LNCM samples. c) High‐resolution transmission electron microscopy (HR‐TEM) images and d) the energy‐dispersive X‐ray spectroscopy profile of the surface of the 20LNM‐ALF_3_‐coated LNCM sample.

**Figure 3:**
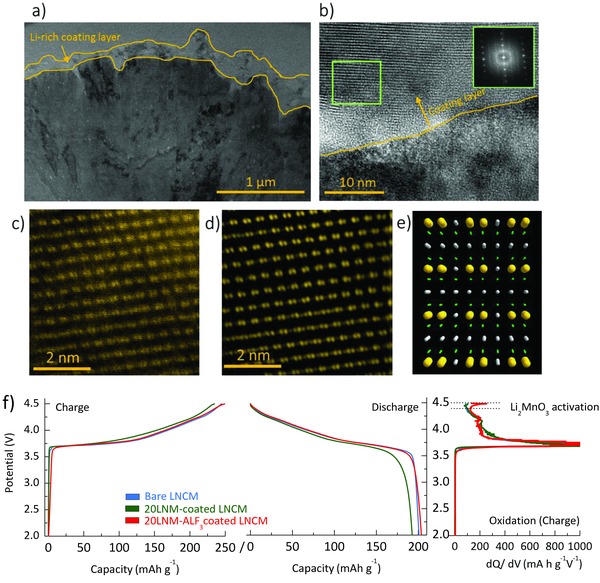
High‐resolution transmission electron micrograph of a) cross‐sectional view and b) the interface between a core and a shell of 20LNM‐ALF_3_‐coated LNCM (the inset figure in panel (b) indicates a pattern of fast Fourier transform of the green square). c) High angle annular dark field (HAADF) and d) the filtered images of the green square region in panel (b). e) Simulated crystal structure of Li_2_MnO_3_ (C2/m) along the rhombohedral [2 1 0] direction (the balls with yellow, green, and grey, respectively, indicate transition metals, oxygen, and lithium ions). f) Profiles of the voltage and differential capacities of the initial cycle of the bare LNCM, 20LNM‐coated LNCM, and 20LNM‐ALF_3_‐coated LNCM samples.

The morphologies of the bare Ni‐rich LiNi_0.7_Co_0.15_Mn_0.15_O_2_ (heareafter referred to as bare LNCM) and LNCM coated with 20 wt% Li‐rich Li_1.2_Ni_0.2_Mn_0.6_O_2_ (LNM) and 1 wt% AlF_3_ (heareafter referred to as 20LNM‐ALF_3_‐coated LNCM) are respectively shown in **Figure** **2**a,b. Figure 2a shows the scanning electron microscope (SEM) image of the 10 μm‐sized bare LNCM sample, made up of 100–200 nm primary particles. After the coating process, small nanoparticles are formed at the surface of the secondary particles (Figure 2b–d). The energy‐dispersive X‐ray spectroscopy data in Figure 2d and Table S1 (Supporting Information) indicate that the shell is composed of Mn‐based Li‐rich and AlF_3_ particles. For a detailed structural analysis of the shell layer, high resolution transmission electron spectroscopy and scanning transmission electron microscopy (STEM) images are shown in **Figure** **3**a–e. In Figure 3a, a thick coating layer of 100–150 nm thick covers the pristine surface. Furthermore, Figure 3b shows the clear interface between the core and the shell, and the pattern obtained by the fast Fourier transform of the marked region in Figure 3b matches that of the monoclinic Li_2_MnO_3_ phase (C2/m).^23^, ^24^ The STEM image (Figure 3c) and its filtered image (Figure 3d) of the shell layer shows the typical transition‐metal arrangement of the Li_1.2_Ni_0.2_Mn_0.6_O_2_ phase (Figure 3e), which include C2/m monoclinic phase and R$\bar{3}$m layered phase.^6^, ^9^ From these results, we can conclude that the bare LNCM is covered by a thick Li_1.2_Ni_0.2_Mn_0.6_O_2_ surface layer with good surface coverage.

Note that the surface coverage of the shell layer on the pristine is important, because the surface coating aims to suppress the direct contact between the bare material and the electrolyte.^10^, ^25^, ^26^ However, most recent studies for the surface coating adapted low coating amounts of below 2 wt% to avoid decease in the the specific capacity, although it is not easy for the low amount of coatings to make an intact coating layer with high coating coverage on active materials.^16^, ^22^, ^26^ On the other hand, our study achieves a full coverage coating by using a high coating amount of 10 wt% without any capacity loss, because the designed shell is a Li‐rich material customized by a unique chemical activation method for the high capacity. (We refer the reader to the Experimental Section for the actual coating amount of Li_1.2−_ *_x_*Ni_0.2_Mn_0.6_O_2_ in the 20LNM‐ALF_3_‐coated LNCM sample). Figrue 3e shows the initial voltage profiles of bare LNCM, 20LNM‐AlF_3_‐coated LNCM, and LNCM coated with 10 wt% LNM (hereafter referred to as 20LNM‐coated LNCM sample). Remarkably, the 20LNM‐AlF_3_‐LNCM sample shows an initial discharge capacity of 203 mA h g^−1^ and a Coulombic efficiency of 81.3%, which are comparable to those of the bare LNCM, even with a high coating amount of 10 wt%. Furthermore, when compared to the 20LNM‐coated LNCM sample, the 20LNM‐ALF_3_‐coated LNCM sample shows a strong charge differential capacity peak and a high initial discharge capacity after AlF_3_ treatment (Figure 3f). Note that the additional AlF_3_ treatment aims the chemical activation that chemically extracts lithium ions from Li‐rich materials without high voltage charge processes (Figure S1, Supporting Information).^6^, ^21^, ^27^, ^28^ Therefore, the unique treatment efficiently activates the surface Li_1.2_Ni_0.2_Mn_0.6_O_2_ layer, which enables the high capacity of the shell layer without severe degradations of Ni‐rich core material caused by the high voltage charge process.^22^

**Figure 4:**
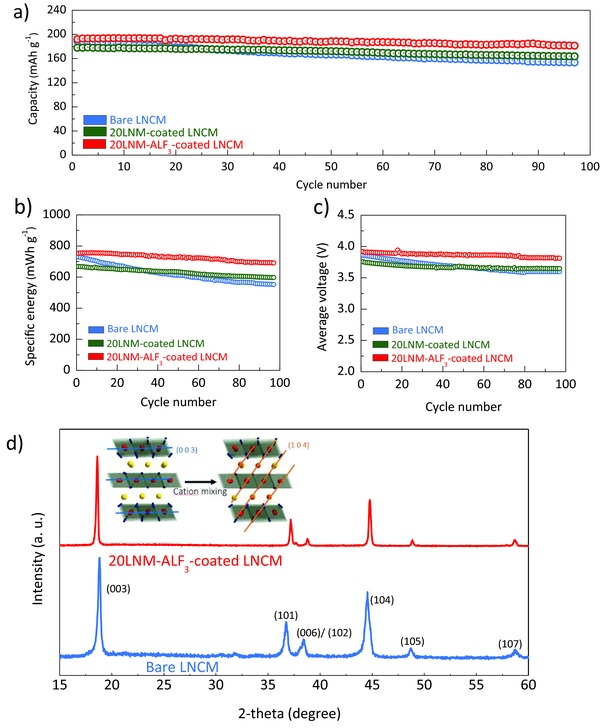
Cycle retention of a) specific capacity, b) gravimetric energy, and c) working voltage as a function of the number of cycles between 2.0 and 4.5 V at a fixed charge/discharge rate of C/3 rate. (1C rate = 200 mA g^−1^). d) Profiles of ex situ X‐ray diffraction patterns of the bare LNCM and 20LNM‐ALF_3_‐coated LNCM samples after the 50 cycles.

**Figure** **4**a highlights the cyclabilities of the bare LNCM, 20LNM‐coated LNCM, and 20LNM‐ALF_3_‐coated LNCM samples. The effect of coating amount on the cyclability is shown in Figure S2 (Supporting Information), and the cyclability of 20LNM‐ALF_3_‐coated LNCM is compared to LNCM coated with AlF_3_ alone (no LNM coating) in Figure S3 (Supporting Information). Remarkably, the 20LNM‐ALF_3_‐coated LNCM sample shows superior cyclability with a capacity retention of 98% during 100 cycles, even when it is cycled with a high state of charge (SOC) between 2.0 and 4.5 V. Considering the superior surface stability of the Li‐rich oxide compared to the Ni‐rich oxide, we can conclude that the observed superior cyclability arises from the stabilization effect of the surface Li_1.2−_ *_x_*Ni_0.2_Mn_0.6_O_2_ layer. Furthermore, because of the chemical activation by the AlF_3_ treatment, the 20LNM‐ALF_3_‐coated LNCM sample shows a high discharge capacity of 190 mA h g^−1^ at C/3 rate, without sacrificing the energy density even with a high coating amount of 10 wt%. Additionally, the 20LNM‐ALF_3_‐coated LNCM sample shows superior specific energy and average working voltage retentions (Figure 4b,c). Considering that the serious drawback of the Mn‐based Li‐rich materials is the working voltage decline, the heterostructure presented here offers the advantage of not only minimizing the shortcomings of the poor energy retention of the Li‐rich oxide but also maximizing the strong points in terms of high capacity and stability.^5^, ^15^

It should be noted that the Ni‐rich layered oxides generally have a structural degradation from R$\bar{3}$m phase to rock salt NiO phase (Fm$\bar{3}$m), that is accelerated by unwanted surface side reactions with electrolyte such as hydrogen fluoride (HF) attack.^10^, ^16^ The degree of formation of the rock salt phase is often evaluated by comparing the X‐ray diffraction (XRD) peak's intensity ratio between the (003) and (104) reflections.^6^ Figure 4d highlights the stabilization effect of the Li‐rich Li_1.2−_ *_x_*Ni_0.2_Mn_0.6_O_2_ coating layer on the structural stability of Ni‐rich active material. Remarkably, 20LNM‐ALF_3_‐coated LNCM shows a high (003)/(104) intensity ratio of 1.3 after 50 cycles, indicating a superior maintenance of the layered structure during cycling, compared to an intensity ratio of 0.98 for the bare LNCM sample, despite both having similar intensity ratios before cycling (Figure S4, Supporting Information). The rock salt phase formation involves the migration of the transition‐metal ions from the transition metal slab to the lithium‐ion slab. Because the NiO formation interrupts lithium‐ion migration as well as reduces the total amount of lithium sites in the cathode material, the phase transition results in severe reversible capacity fade.^29^ As mentioned earlier, the phase transition starts at the surface and then expends to the bulk.^6^, ^11^, ^13^, ^20^ Therefore, the structural stability of the active material is directly affected by the surface stability. In this regard, the superior electrochemical performance of the heterostructured sample is due to the improved surface stability imparted by the shell layer.^10^, ^25^

**Figure 5:**
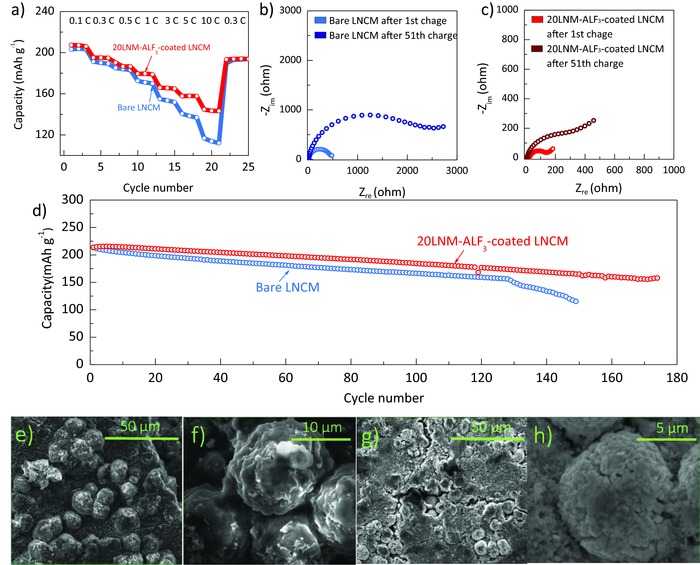
a) Rate capabilities of the bare LNCM and 20LNM‐ALF_3_‐coated LNCM samples with increasing C rates from 0.1 to 10 C rate between 2.0 and 4.5 V at 25 °C. Ex situ electrochemical impedance spectroscopy (EIS) results of b) the bare LNCM and c) 20LNM‐ALF_3_‐coated LNCM samples after 1st charge and 51th charge process to 4.5 V. d) Cycle performance between 2.0 and 4.5 V at 1C rate and 55 °C. Scanning electron images of the e,f) bare LNCM and g,h) 20LNM‐ALF_3_‐coated LNCM samples after the cycle test in panel (d).

The surface stability of the coating layer also leads to improved rate capability of the 20LNM‐ALF_3_‐coated LNCM sample (**Figure** **5**a). Recently, many studies have reported that the rate performance of active materials depends highly on their surface stability because the surface instability continuously leads to the formation of side reactions with the electrolyte and structural reorganizations, in turn resulting in a severe degradation of lithium‐ion and electronic mobilities.^6^, ^13^, ^30^ Therefore, although it is well known that the surface Li_1.2_Ni_0.2_Mn_0.6_O_2_ phase has a lower lithium‐ion and electronic conductivity than the bare LiNi_0.7_Co_0.15_Mn_0.15_O_2_ phase, the coated sample shows better rate capability. In order to verify the stabilization effects of the designed coating on the Ni‐rich material surface, ex situ electrochemical impedance spectroscopy (EIS) test was conducted. (Figure 5b,c and Figure S5, Supporting Information). A large impedance of the bare LNCM sample is observed after the first charge process to 4.5 V, compared to that of the 20LNM‐ALF_3_‐coated LNCM sample, and the result is coincident with the rate capability test in Figure 5a. It should be noted that the high SOC to 4.5 V causes substantial surface degradations on Ni‐rich materials such as thick SEI layer formation and phase transition to rock salt phase, which leads to severe rise in impedance in terms of lithium‐ion and electron mobility. Furthermore, after 50 cycles, the uncoated bare LNCM sample shows dramatically increased impedance for charge transfer, as indicated by a huge semicircle of about five times larger in diameter than that of the first cycle, compared to that of the 20LNM‐ALF_3_‐coated LNCM sample. The continuous increase in resistance results in a severe voltage drop of the bare LNCM sample, as shown in Figure S6 (Supporting Information). The ex situ EIS results support that the designed surface layer can alleviate the surface instability of the Ni‐rich layered oxide and suppress the continuous formation of thick SEI layer during electrochemical cycling.

Figure 5d highlights the cyclability at elevated temperature (55 °C) of the 20LNM‐ALF_3_‐coated LNCM sample compared to that of the bare LNCM sample. The 20LNM‐ALF_3_‐coated LNCM sample shows superior cyclability with 87% of capacity retention (from 213 to 185 mA h g^−1^) during 100 cycles, compared to 77% for the bare LNCM sample (from 213 to 163 mA h g^−1^).^16^, ^31^ Oddly, the bare LNCM sample shows dramatic capacity fade after the 125th cycle. To verify the origin of the severe capacity drop of the bare LNCM sample, ex situ SEM was conducted. Thick SEI layers are observed on the surface of the bare LNCM sample after the cycle test at 55 °C (Figure 5e,f), while the 20LNM‐ALF_3_‐coated LNCM sample shows a clear surface of the secondary particles (Figure 5g,h). The formation of thick SEI layer at the surface of the bare LNCM leads to its poor rate capability and severe capacity drop during cycling. Furthermore, structural collapse of active materials is often accelerated by the surface side reactions with electrolyte.^6^, ^13^, ^16^, ^32^ As a result, the bare LNCM sample shows severe phase distortion after the cycling at 55 °C (Figure S7, Supporting Information). The severe phase transition of the bare LNCM sample results in not only poor surface lithium‐ion conductivity and increased charge‐transfer resistance but also decreases the electrochemically active lithium sites, in turn resulting in a dramatic capacity drop. The rate capabilities of bare LNCM and 20LNM‐AlF_3_‐coated LNCM samples at 60 °C are also highlighted in Figure S9 (Supporting Information). These results support that the designed Li‐rich (Li_1.2−_ *_x_*Ni_0.2_Mn_0.6_O_2_) layer enhances the surface electrochemical stability of the Ni‐rich sample, which leads to the superiority of the coated sample in terms of cyclability during cycling at 55 °C as well as 25 °C.

**Figure 6:**
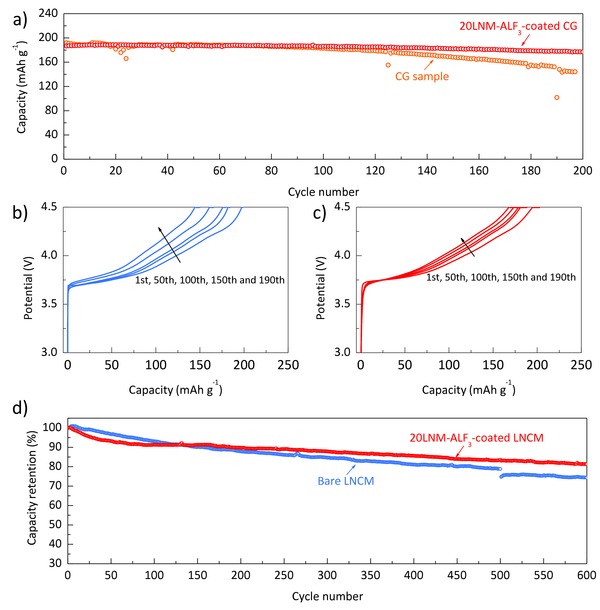
a) Cycle performance of the concentration‐gradient sample (CG) and Li_1.2−_ *_x_*Ni_0.2_Mn_0.6_O_2‐_coated CG sample (20LNM‐ALF_3_‐coated CG) at C/3 rate. Charge voltage profiles of b) CG and c) 20LNM‐ALF_3_‐coated CG samples during cycling at C/3 rate. d) Full‐cell cyclability of the bare LNCM and 20LNM‐ALF_3_‐coated LNCM samples between 2.5 and 4.4 V at 1C rate and 25 °C.

To show the superiority of the stabilization effect of the modified Li‐rich shell (Li_1.2−_ *_x_*Ni_0.2_Mn_0.6_O_2_), the surface modification was applied onto a Mn concentration‐gradient (CG) Ni‐rich sample, which has been suggested to solve the surface chemical instability of the Ni‐rich oxide;^33^ the CG sample consists of a Ni‐rich core and a Mn‐rich surface, with the Ni content gradually decreasing from the interir to the esterior. The surface‐modified CG sample (20LNM‐ALF_3_‐coated CG) shows better long‐term cycleability with a superior capacity retention of 95% compared to uncoated CG sample and 20LNM‐ALF_3_ LNCM sample (**Figure** **6**a–c and Figure S10, Supporting Information). The cycle results indicate that the modified Li‐rich layer effectively improves the surface stability of the Ni‐rich materials compared to the CG sample. Considering the increase in resistance during cycling, the 20LNM‐ALF_3_‐coated CG sample shows stable charge voltage profiles during 200 cycles, while the uncoated CG sample undergoes severe resistance increase. Remarkably, the 20LNM‐ALF_3_‐coated LNCM sample shows a notable full‐cell performance, when the full‐cell was assembled with a natural graphite anode (Figure 6d and Figure S11, Supporting Information). Compared to a full‐cell data of the bare LNCM sample, the 20LNM‐ALF_3_‐coated LNCM sample has an improved cycle retention of 82% during 600 cycles at 1C rate.

## 3 Conclusion

We show that a heterostructure composed of Ni‐rich core layered oxide core and a Li‐rich Li_1.2−_ *_x_*Ni_0.2_Mn_0.6_O_2_ shell layer overcomes the critical drawbacks of both materials: the surface electrochemical instability with electrolyte of the core material as well as the voltage decline problem of the shell layer. Even though a high coating amount of 10 wt% was applied for a high‐coverage coating, the coated material shows a high reversible capacity of 200 mA h g^−1^ due to a unique surface treatment that aims at a chemical activation of the surface Li_1.2_Ni_0.2_Mn_0.6_O_2_ layer. As a consequence, the customized surface Li_1.2−_ *_x_*Ni_0.2_Mn_0.6_O_2_ layer alleviates the surface instability of the core LiNi_0.7_Co_0.15_Mn_0.15_O_2_, which leads to superiority in terms of rate capacity and cyclability at 55 °C as well as 25 °C. The study demonstrates that the Li‐rich shell layer can stabilize the surface of the Ni‐rich layered oxide when they are cycled below 4.5 V.

## 4 Experimental Section

*Preparation of the Bare LNCM Material*: To prepare the hydroxide precursor Ni_0.7_Co_0.15_Mn_0.15_(OH)_2_, NiSO_4_⋅6H_2_O, CoSO_4_⋅7H_2_O, and MnSO_4_⋅H_2_O were dissolved in a molar ratio of Ni:Co:Mn = 70:15:15 in distilled water at a combined concentration of 1 m. A 2.5 L continuously stirring tank reactor (CSTR) was used for the coprecipitation reaction, and an appropriate amount of saturated NH_4_OH and KOH were added in order to reach the initial condition. Subsequently, the mixed‐metal solution was fed separately into the CSTR along with 2 m KOH aqueous solution under N_2_ atmosphere. During the reaction, the pH ( = 11), the amount of NH_4_OH added as a chelating agent, and the temperature (50 °C) were monitored and adjusted carefully. The total feed rate was regulated to assure an average residence time of 4–12 h in the reactor. The coprecipitated particles were filtered, washed, and dried at 120 °C under vacuum for 24 h to obtain the Ni_0.7_Co_0.15_Mn_0.15_(OH)_2_ precursor. The prepared precursor was mixed with LiOH⋅H_2_O at a molar ratio of 1:1.03 and preheated at 500 °C for 5 h and then calcined at 800 °C for 15 h to obtain the Ni‐rich layered oxide LiNi_0.7_Co_0.15_Mn_0.15_O_2_ (denoted as the bare LNCM).

*Coating Process*: The as‐prepared bare LNCM sample was mixed with the coating precursor consisting of a desired amount of Li(CH_3_COO)⋅2H_2_O, Ni(CH_3_COO)⋅4H_2_O, Mn(CH_3_COO)⋅4H_2_O in ethanol solvent. After stirring for 30 min, the coating solution was dried at 120 °C for 10 h in the air. The obtained material was then fired at 700 °C for 5 h. For the chemical activation of the single‐coated sample (denoted as 20LNM‐coated LNCM), the as‐prepared single coated powder was immersed in an ethanol solution consisting of NH_4_F and Al(NO_3_)_3_⋅9H_2_O and dried at 120 °C for 10 h. The obtained powder was then heated at 400 °C for 5 h in air. After the heating process, the resulting powder (denoted as 20LNM‐ALF_3_‐coated LNCM) was sieved to remove the large coated, aggregated particles. As a result, the actual coating amount of Li_1.2−_ *_x_*Ni_0.2_Mn_0.6_O_2_ on 20LNM‐ALF_3_‐coated LNCM sample was 10 wt% (Figure S9 and Table S2, Supporting Information).

*Structural Characterization*: Powder XRD (MiniFlex 600, Rigaku) with CuKα radiation was employed to structurally characterize the samples. The surface morphologies of the samples were observed by SEM (Quanta 650 ESEM, FEI). A field‐emission electron microscope (JEM‐2100F, JEOL) was used to identify the surface morphologies of the 20LNM‐ALF_3_‐coated LNCM sample and the formation of surface Li_1.2_Ni_0.2_Mn_0.6_O_2_ layer at atomic resolution.

*Electrochemical Tests*: The electrochemical performances of the different samples were assessed with a coin‐type (2032R) half‐cell with a lithium‐metal anode. The electrolyte consisted of 1 m LiPF_6_ in ethylene carbonate (EC)/dimethyl carbonate (1:1 vol%). The cathode electrode consisted of Super P carbon black, polyvinylidene fluoride binder, and active material, in a weight ratio of 1:1:8 with active material loaded at 4.0–4.5 mg cm^−2^. A full‐cell was assembled with natural graphite as anode (the negative to positive electrode N/P ratio was fixed at 1.1) and cycled at 1C rate under 25 °C for 600 cycles. The full cell used an electrolyte solution of 1.2 m LiPF_6_ in EC/ethyl methyl carbonate (3:7 vol%) with 2 vol% of vinylene carbonate additive.

## Supporting information

As a service to our authors and readers, this journal provides supporting information supplied by the authors. Such materials are peer reviewed and may be re‐organized for online delivery, but are not copy‐edited or typeset. Technical support issues arising from supporting information (other than missing files) should be addressed to the authors.

Supplementary
