# Supplementary material for: High‐Performance Heterostructured Cathodes for Lithium‐Ion Batteries with a Ni‐Rich Layered Oxide Core and a Li‐Rich Layered Oxide Shell
Source: Adv Sci (Weinh). 2016 May 30;3(11):1600184. doi: 10.1002/advs.201600184 (PMC5111731; doi:10.1002/advs.201600184)
Supplement: Supplementary file 1 — Supplementary [file ADVS-3-0l-s001.pdf]

Copyright WILEY-VCH Verlag GmbH & Co. KGaA, 69469 Weinheim, Germany, 2016.

ADVANCED SCIENCE
Open Access

Supporting Information

for *Adv. Sci.,* DOI: 10.1002/advs.201600184

High-Performance Heterostructured Cathodes for Lithium-Ion Batteries with a Ni-Rich Layered Oxide Core and a Li-Rich Layered Oxide Shell

*Pilgun Oh, Seung-Min Oh, Wangda Li, Seunjun Myeong, Jaephil Cho, and Arumugam Manthiram**

Copyright WILEY-VCH Verlag GmbH & Co. KGaA, 69469 Weinheim, Germany, 2013.

Supporting Information

High-performance Heterostructured Cathodes for Lithium-ion Batteries with a Ni-rich Layered Oxide Core and a Li-rich Layered Oxide Shell

*Pilgun Oh, Seung-Min Oh, Wangda Li, Seunjun Myeong, Jaephil Cho, and Arumugam Manthiram**

Table S1. Quantitative analysis of energy-dispersive X-ray spectroscopy (EDS) data in Figure 2d (20LNM-ALF$_3$-coated LNCM sample)

**Element**	**O**	**F**	**Al**	**Mn**	**Co**	**Ni**	**Total:**
At. %	58.4	4.6	1.8	23.2	4.5	7.3	100.0

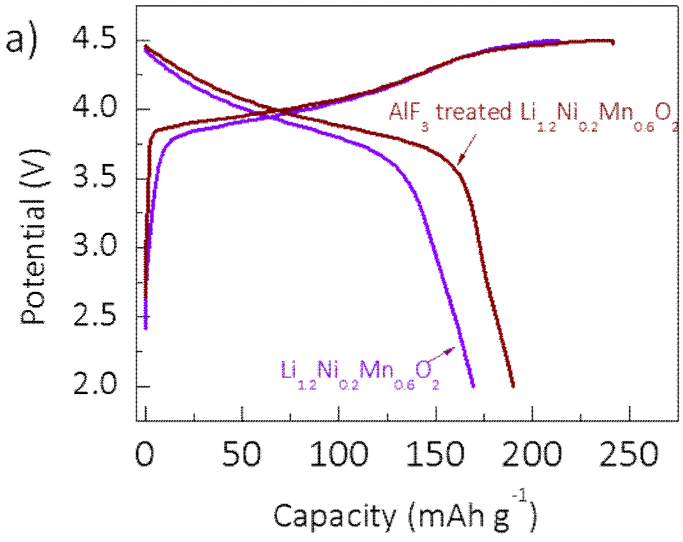

b)

	**Li**	**Ni**	**Mn**	**Li/TM**
**$Li_{1.2}Ni_{0.2}Mn_{0.6}O_2$**	5.94	1.00	3.00	1.50
**AlF_3-treated sample**	5.66	1.00	3.00	1.42

Figure S1. (a) Voltage profiles and (b) ICP data of $Li_{1.2}Ni_{0.2}Mn_{0.6}O_2$ and AlF_3-treated $Li_{1.2}Ni_{0.2}Mn_{0.6}O_2$ sample.

To verify the AlF_3 treatment effect on the surface $Li_{1.2}Ni_{0.2}Mn_{0.6}O_2$ layer of 20LNM-ALF$_3$-coated LNCM sample, the AlF_3 treatment was conducted on $Li_{1.2}Ni_{0.2}Mn_{0.6}O_2$ powder. The AlF_3-treated sample shows a higher charge and discharge capacity than the pristine sample (AlF_3-untreated sample). Recently, Cho et al. reported that chemical treatments using acid solvents can extract lithium ions from Li_2MnO_3 phase of Mn-based Li-rich materials, and the chemical treatment helps the activation of Li_2MnO_3 phase.[1,2] In their result, the Li_2MnO_3 phase was fully activated after the chemical activation although they used semi-micron sized particles of $Li_{1.2}Ni_{0.2}Mn_{0.6}O_2$. To support the evidence of chemical activation of Li_2MnO_3 phase on 20LNM-ALF$_3$-coated LNCM, a quantitative analysis with inductively coupled plasma (ICP) analysis was conducted. Remarkably, the AlF_3-treated sample shows lower lithium concentration than the pristine sample. It means the AlF_3 treatment extracts lithium ions from the surface of the $Li_{1.2}Ni_{0.2}Mn_{0.6}O_2$ layer of 20LNM-ALF$_3$-coated LNCM. Then, this chemical activation leads to a higher reversible capacity of the 20LNM-ALF$_3$-coated LNCM sample compared to the 20LNM-coated LNCM sample.

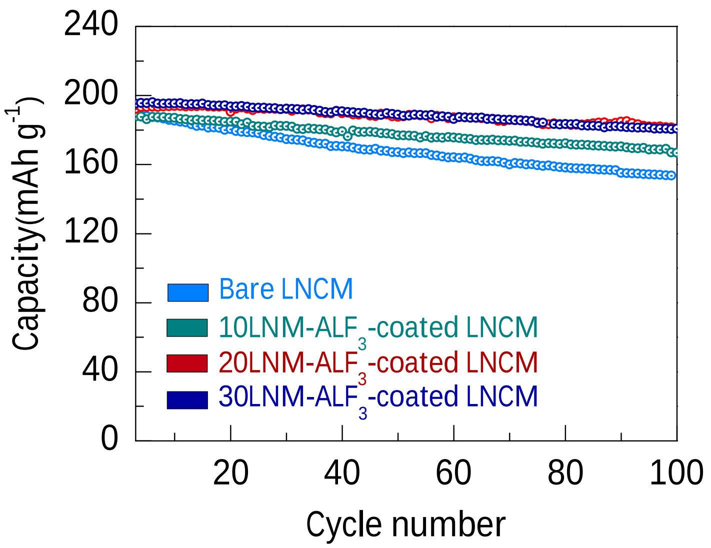

Figure S2. Cycle performance of the bare LNCM and coated samples with various coating amounts from 10 to 30 wt. % during 100 cycles at C/3 rate.

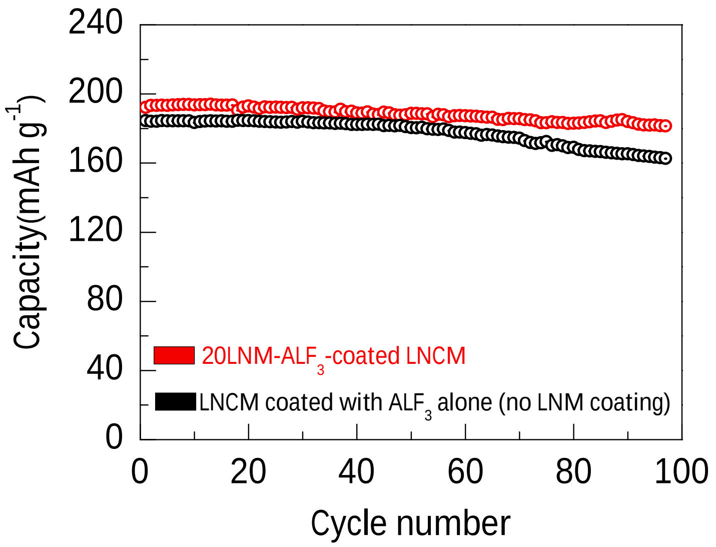

Figure S3. Cyclability of 20LNM-ALF_3-coated LNCM and LNCM coated with AlF_3 alone.

To know the actual coating effect of $Li_{1.2-x}Ni_{0.2}Mn_{0.6}O_2$, single AlF_3 treatment was conducted on the bare LNCM sample. As a result, the LNCM coated with AlF_3 sample shows lower reversible capacity and poor cyclability compared to the 20LNM-ALF_3-coated LNCM sample.

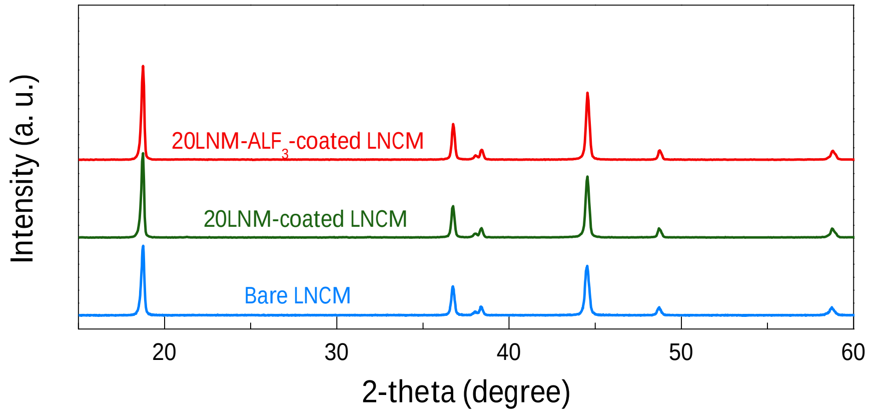

Figure S4. X-ray diffraction (XRD) data of the bare LNCM, 20LNM-coated LNCM, and 20LNM-ALF_3-coated LNCM samples before cycling.

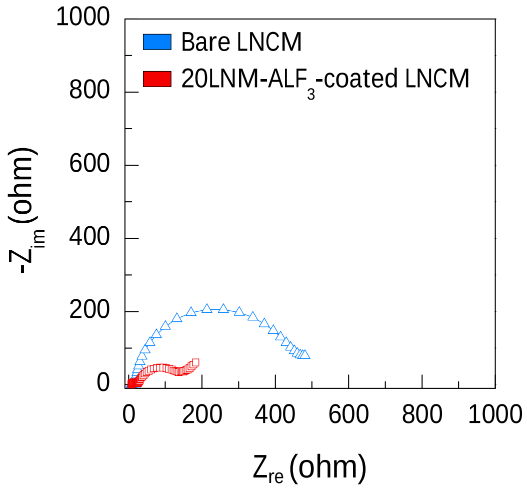

Figure S5. Ex-situ electrochemical impedance spectroscopy (EIS) results of the bare LNCM and 20LNM-ALF$_3$-coated LNCM samples after the 1st charge process to 4.5 V at 0.1 C rate.

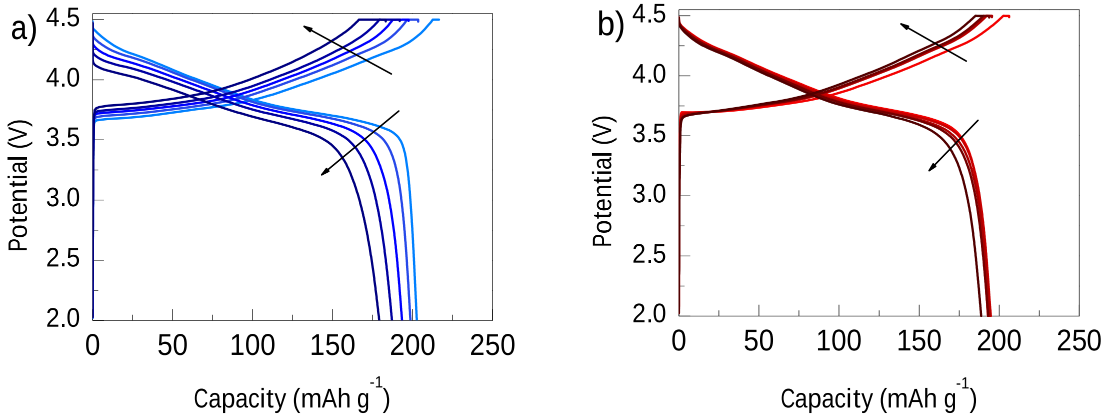

Figure S6. Voltage-capacity profiles of the (a) bare LNCM and (b) 20LNM-ALF$_3$-coated LNCM samples during 50 cycles at C/3 rate.

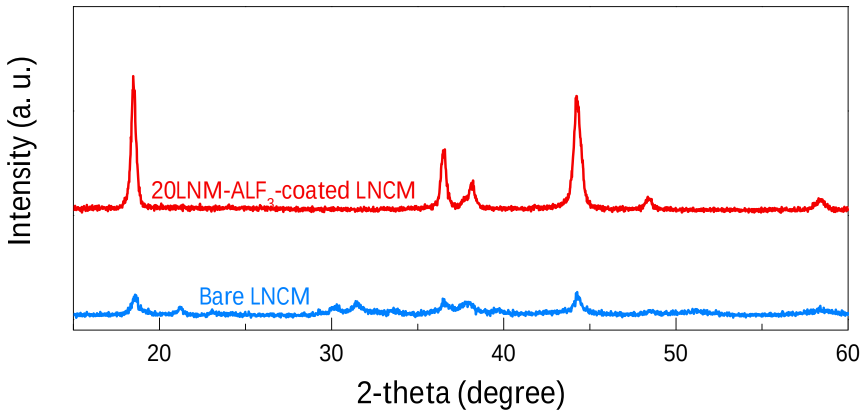

Figure S7. *Ex-situ* XRD of the bare LNCM and 20LNM-ALF$_3$-coated LNCM samples after the cycle test of Figure 5d.

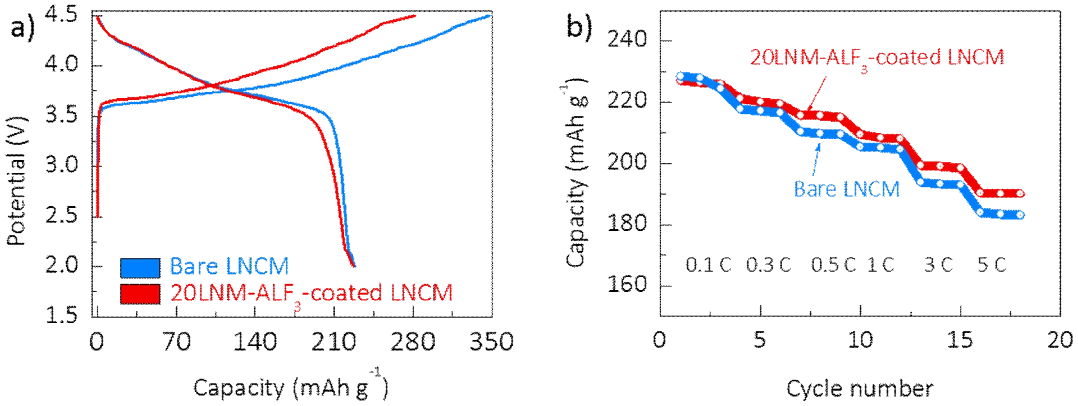

Figure S8. (a) Voltage profiles of the bare LNCM and 20LNM-ALF_3-coated LNCM samples between 2.0 and 4.5 V at 60 °C. (b) Rate capabilities of the bare LNCM and 20LNM-ALF_3-coated LNCM samples with increasing C rates from 0.1 to 5C rate at 60 °C.

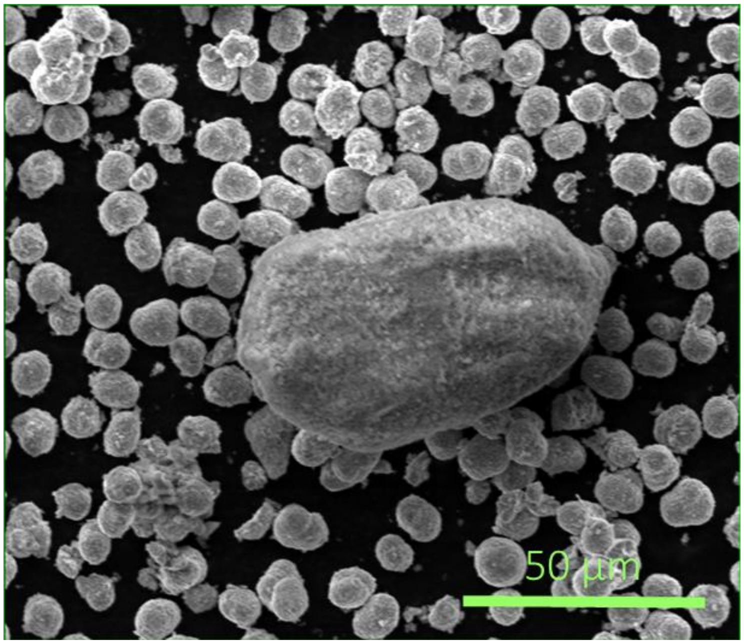

Figure S9. SEM image of the 20LNM-coated LNCM sample before sieve process.

Table S2. Inductively coupled plasma-atomic emission spectroscopy (ICP- AES) data of the 20LNM-ALF$_3$-coated LNCM

	**Relative molar ratio**	**Lithium/ Transition metal**
**Li**	1.69	1.08
**Ni**	1.00	
**Mn**	0.32	
**Co**	0.22	
**Al**	0.01	

Large coating materials and aggregated particles were removed by a sieving process. As a result, the ICP data of the 20LNM-ALF$_3$-coated LNCM sample shows a molar ratio of 1.08 between lithium and transition metals, which indicates that the resulting material's chemical formula is $0.1Li_{1.2}Ni_{0.2}Mn_{0.6}O_2$-$0.9LiNi_{0.7}Co_{0.15}Mn_{0.15}O_2$ and the actual coating amount of $Li_{1.2}Ni_{0.2}Mn_{0.6}O_2$ is about 10 wt. %.

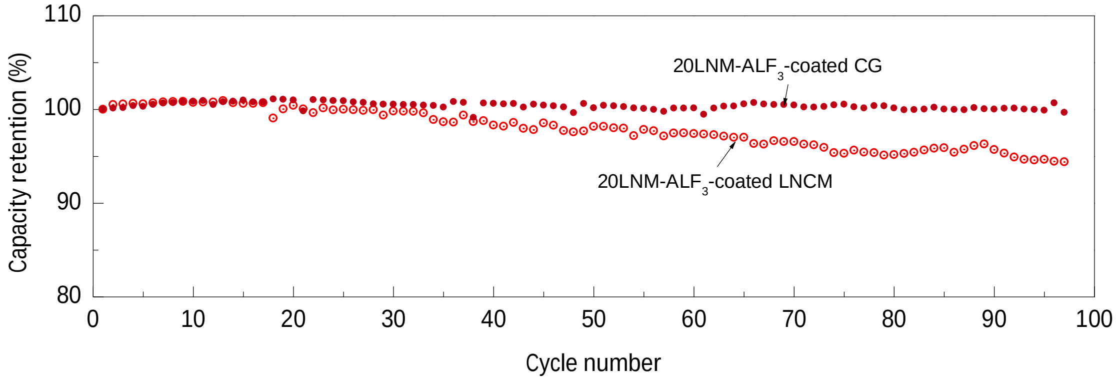

Figure S10. Discharge capacity retention of 20LNM-ALF$_3$-coated LNCM and 20LNM-ALF$_3$-coated CG samples during 100 cycles at C/3 rate.

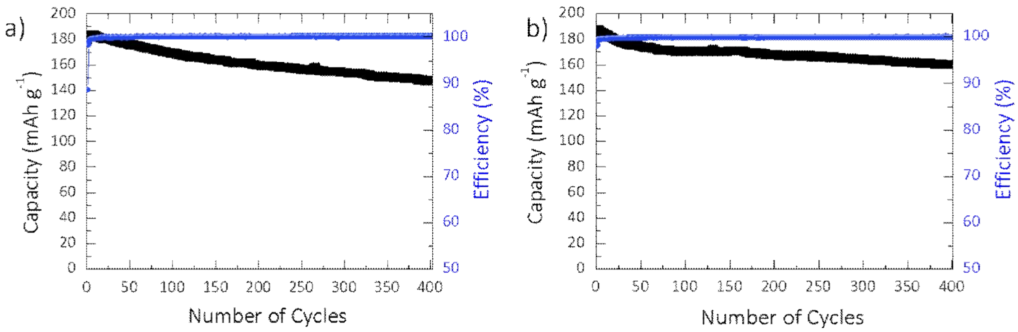

Figure S11. Full cell data of the (a) bare LNCM and (b) 20LNM-ALF$_3$-coated LNCM samples assembled with natural graphite anode between 2.4 and 4.4 V at 1C rate. The negative to positive electrode (N/ P) ratio was fixed at 1.1

REFERENCES

[1] P. Oh, M. Ko, S. Myeong, Y. Kim , J. Cho, *Adv. Energy Mater.*, **2014,** 4, 9.

[2] P. Oh, S. Myeong, W. Cho, M. J. Lee, M. Ko, H. Y. Jeong , J. Cho, *Nano Lett.*, **2014,** 14, 5965.
